# Progressively Unsupervised Generative Attentional Networks with Adaptive Layer-Instance Normalization for Image-to-Image Translation

**DOI:** 10.3390/s23156858

**Published:** 2023-08-01

**Authors:** Hong-Yu Lee, Yung-Hui Li, Ting-Hsuan Lee, Muhammad Saqlain Aslam

**Affiliations:** 1 Department of Computer Science and Information Engineering, National Central University, Taoyuan 32001, Taiwan; sam3u7858@gmail.com (H.-Y.L.); s109522056@g.ncu.edu.tw (T.-H.L.); 2 AI Research Center, Hon Hai Research Institute, Taipei 114699, Taiwan; yunghui.li@foxconn.com

**Keywords:** anime, cartoon styles, generative adversarial networks, image-to-image translation, style transfer

## Abstract

Unsupervised image-to-image translation has received considerable attention due to the recent remarkable advancements in generative adversarial networks (GANs). In image-to-image translation, state-of-the-art methods use unpaired image data to learn mappings between the source and target domains. However, despite their promising results, existing approaches often fail in challenging conditions, particularly when images have various target instances and a translation task involves significant transitions in shape and visual artifacts when translating low-level information rather than high-level semantics. To tackle the problem, we propose a novel framework called Progressive Unsupervised Generative Attentional Networks with Adaptive Layer-Instance Normalization (PRO-U-GAT-IT) for the unsupervised image-to-image translation task. In contrast to existing attention-based models that fail to handle geometric transitions between the source and target domains, our model can translate images requiring extensive and holistic changes in shape. Experimental results show the superiority of the proposed approach compared to the existing state-of-the-art models on different datasets.

## 1. Introduction

In recent years, generative adversarial networks (GANs) have made significant progress in image-to-image translation. Researchers in machine learning and computer vision have given this topic considerable attention because of the wide range of practical applications available [1,2]. These include image inpainting [3,4], colorization [5,6], super-resolution [7,8], and style transfer [9,10]. Image-to-image translation refers to a category of vision and graphics problems in which the goal is to learn the mapping between an input image (source domain) and an output image (target domain) from a set of aligned image pairs [11]. In the case of portrait stylization, various methods have been explored, such as self-to-anime [1] and cartoon [12]. There are, however, many tasks that will not offer paired training data. When paired data are provided, the mapping model can be trained using a conditional generative model [13,14,15] or a simple regression model [5,16,17] in a supervised manner.

Various works [18,19,20,21,22,23,24,25] have successfully translated images in unsupervised settings without available paired data by assuming shared latent space [22] and cycle consistency assumptions [11,21]. Nevertheless, supervised approaches require paired datasets for training, which can be laborious and expensive, if possible, to prepare manually. In contrast, unsupervised methods need a large volume of unpaired data and frequently need help to reach stable training convergence and generate high-resolution results [26].

Previous techniques have shortcomings despite their progress and benefits and often fail to meet challenging tasks, especially when the target images have multiple instances to be translated [27] or the shape of the target instances has drastically changed [11]. For example, they are efficient for style transfer tasks that map local textures such as photo2vangogh and photo2portrait. However, they are ineffective for image translation tasks with extensive shape transformations, such as selfie2anime and cat2dog, in wild images. As a result, pre-processing measures such as image cropping and alignment can significantly prevent these difficulties by limiting the complexity of data distributions [1,2]. Further, current methods like DRIT [28] cannot produce the coveted results for both image translation that preserves appearance (such as horse2zebra) and image translation that transforms shape (such as cat2dog) due to the fixed network structure and hyperparameters. There is a need to adjust the network architecture or hyperparameters for each dataset.

In 2014, Ian Goodfellow et al. [29] introduced generative adversarial networks (GANs), which can solve image-to-image problems, including anime face style transfer. A study published in 2017 found that Pix2Pix [13] and CycleGAN [11] are the two primary GAN-based approaches that can successfully address image-to-image problems. CartoonGAN [30] was introduced in 2018 as an upgrade of Pix2Pix, specializing in the cartoon sector. Nevertheless, all the earlier methods merely transfer textures. Junho Kim et al., therefore, presented U-GAT-IT [1], a technique based on CycleGAN that can handle both texture and geometry transfer. However, in the generated image, geometry factors differ dramatically from a human face image. Consequently, the output does not maintain the input signature.

This paper proposes Progressive U-GAT-IT (PRO-U-GAT-IT), a novel framework for unsupervised image-to-image translation tasks, which incorporates an attention module and learnable normalization function in an end-to-end strategy. Based on the attention map obtained by the auxiliary classifier, our model guides the translation so that it focuses on more essential regions and disregards minor areas by distinguishing between the source and target domains. Furthermore, these attention maps are embedded in the generator and discriminator to emphasize relevant critical areas, thereby enabling shape transformation. For example, a generator’s attention map focuses on regions that distinguish between the two domains. In contrast, a discriminator’s attention map assists in fine-tuning by concentrating on the difference between real and fake images. Additionally, we discovered that the selection of the normalization function substantially influences the quality of the transformed outcomes for various datasets with varying degrees of shape and texture changes. Furthermore, earlier approaches have limitations, including blurry results, unstable training, low resolutions, and limited variation. Moreover, high-resolution images are difficult to generate because their higher resolution makes them easily distinguishable from training images. Finally, due to memory constraints, large resolutions also require smaller mini-batches, compromising training stability. Nevertheless, recent improvements in the resolution and quality of images produced by generative methods, particularly GANs, have been observed.

The contributions of our work are summarized as follows:

- We propose a framework that improves the image-to-image translation model through a progressive block-training approach. This novel technique allows for the acquisition of distinct features during various training phases, leading to several notable advantages. These include reduced VRAM usage, accelerated training speed on par with or surpassing other methods when using the same device, and the ability to achieve successful image translation at higher resolutions.
- Furthermore, we propose a novel research field that emphasizes the exploration and refinement of progressive image-to-image translation techniques. Our aim is to enhance both the quality of results and the overall efficiency of image-to-image translation models.

## 2. Related Work

### 2.1. Generative Adversarial Networks (GANs)

GANs [29] are persuasive generative models that have attained pleasing results in various applications of computer vision tasks such as super-resolution imaging [31] and image [32] and video generation [33]. Karras et al. proposed a method based on a simple progressive growing of GANs [34] to synthesize largely (for example, 256 × 256) realistic images in an unconditional environment. In a GAN framework, the goal of the generative model is to fool a discriminator by generating fake images, whereas that of the discriminative model is to differentiate between the generated samples and actual samples. Furthermore, for generating meaningful images that satisfy user needs, Conditional GANs (CGANs) [35,36] add additional information, such as discrete labels [19,37], object key points [38], human skeletons [39,40], and semantic maps [36,41,42], to assist in the image generation process.

### 2.2. Image-to-Image Translation

Convolutional neural networks (CNNs) have been used to learn a translation function for image-to-image translation. The task is to find a mapping between a source and a target domain. The models used in the early methods utilize a supervised framework, where the model identifies pairs of examples, for instance, by employing a conditional GAN to determine the mapping function [13,15,20]. Philip Isola et al. proposed Pix2pix [13], which is a conditional framework that uses a CGAN to determine a mapping function for input-to-output images. Wang et al. proposed Pix2pixHD [15], a high-resolution photo-realistic image-to-image translation method that can be applied to produce photo-realistic interpretations of semantic label maps. In addition, a similar approach has been implemented for several other tasks, including the generation of hand gestures [39]. However, many real-world tasks encounter the issue of having fewer or no paired input-output samples available. The problem of image-to-image translation becomes ill-posed in the absence of paired training data.

Several methods that perform unpaired image-to-image translations have recently been proposed to address this limitation, producing remarkable results. These methods are essential for applications that lack or cannot obtain paired data and determine the mapping function without requiring paired training data. In particular, CycleGAN [11] learns to map between two domains of images rather than pairs of images. Besides CycleGAN, many other variants of GAN have been proposed [18,21,25,43,44,45] to deal with the cross-domain problem. However, the drawback of these models is that they can be easily affected by undesired content and cannot identify the most discriminative semantic information about images during the translation phase.

Several works have employed an attention mechanism to alleviate these shortcomings. Many applications in computer vision have successfully implemented attention mechanisms, including depth estimation [46], which allows the models to concentrate on a significant part of the input. In some recent studies, attention modules have been used unsupervised to pay attention to the region of interest (ROI) in the image translation task, which can be divided into two categories. The first category involves providing attention using additional data. For example, Liang et al. introduced ContrastGAN [47], which utilizes the object mask annotations from every dataset as additional input data.

Furthermore, Mo et al. proposed InstaGAN [2], which combines instance information (such as object segmentation masks) to enhance multi-instance transfiguration. Another method involves training segmentation or an attention model to produce attention maps and adapt them to the system. For example, Chen et al. [8] generated attention maps using an additional attention network to highlight objects of interest more. Kazaniotis et al. presented ATAGAN [48], which generates attention maps using a teacher network. A new module was proposed by Yang et al. [49] that predicts an attention map to guide the image translation method. Kim et al. [1] introduced the U-GAT-IT model to circumvent the challenge of geometry transfer. The key objective of the model is to pay more attention to the regions that contain distinctive anime-style representations. For this purpose, an auxiliary classifier is used to generate attention masks. In a study by Mejjati et al. [50], attention mechanisms were implemented with generators, discriminators, and two other attention networks.

## 3. Methodology

**Figure 1:**
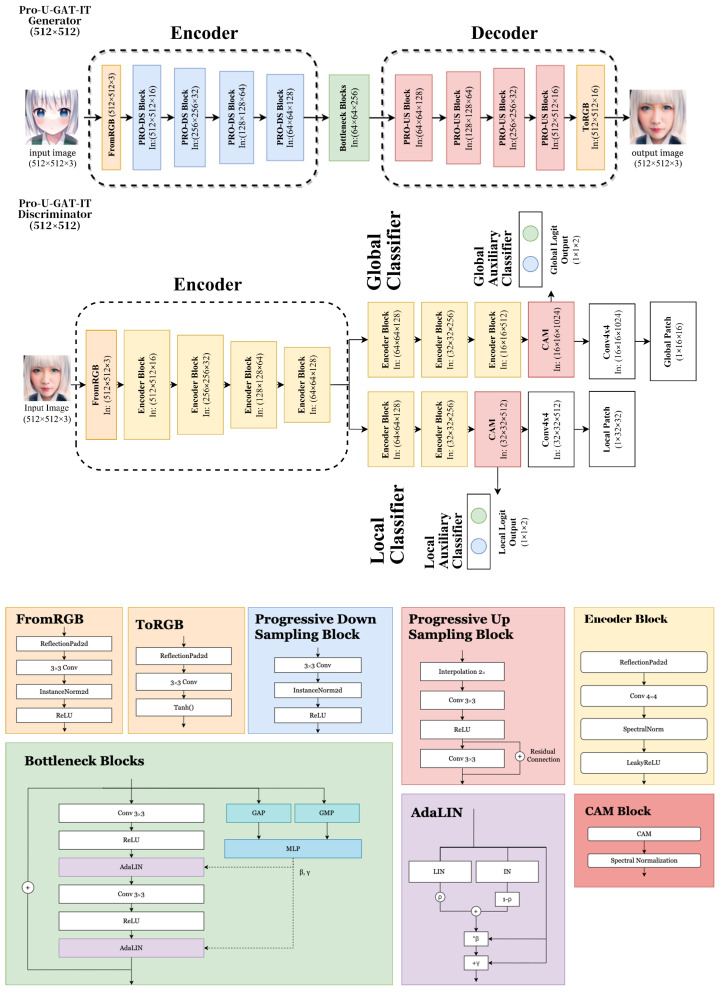
The proposed PRO-U-GAT-IT architecture.

We propose a translation model called PRO-U-GAT-IT, as shown in Figure 1. Based on the U-GAT-IT architecture, the model implements progressive blocks, such as Progressive Downsampling (PRO-DS) blocks and Progressive Upsampling (PRO-US) blocks, inspired by Progressive GANs [34]. The progressive blocks are trained to learn features from low resolution to high resolution by gradually adding new progressive blocks to the network, helping to generate larger images and to enhance quality.

### 3.1. Generator

A generator comprises a series of PRO-DS blocks as the encoder, bottleneck blocks, and PRO-US blocks as the decoder. The encoder and the decoder are like a mirror, synchronizing the number of progressive blocks. Such an increase allows the model to learn from large outlines of the image to details of the objects, which learn different scale features separately. Progressive training generates high-resolution images without losing image details, improves training speed, and reduces model consumption through training the model. The model does not have to be learned from scratch when the resolution is enhanced, saving time for generating high-resolution images. Moreover, we modified the PRO-US block by adding a residual connection between two convolution layers, which improves image quality.

### 3.2. Discriminator

Similar to the U-GAT-IT model, the discriminator consists of an encoder, a classifier, and an auxiliary classifier. We progressively add the encoder blocks before reaching the size of the 64 × 64 × 128 encoder block to accommodate different input image sizes, depending on the generator’s output. In addition, we follow the U-GAT-IT approach, adopting two different scales of patch for the discriminator to classify the local (1 × 32 × 32) and global (1 × 16 × 16) receptive fields.

### 3.3. Loss Function

**Cycle Loss:** Without paired data, we apply a cycle consistency constraint to the generator to minimize the model collapse problem by forcing $G_{s\to t}$ and $G_{t\to s}$ to contradict each other. The cycle consistency loss calculates the difference between the image $x\in X_{s}$, and the image transformed into the domain *t* and back into the domain *s*.

(1) $$L_{Cycle}^{s\to t}=E_{x}{}_{∼}{}_{X}{}_{s}[\;|x-G_{t\to s}\left(\;G_{s\to t}\left(\;x\right)\;\right)\;{|}_{1}]\;$$

$$L_{C}ycle=L_{Cycle}^{s\to t}+L_{Cycle}^{t\to s}$$

**Identity Loss:** Identity loss helps preserve the consistency of input and output color composition by enforcing identity mapping when real samples of the target domain are given as the input to the generator. For example, given the input $x\in X_{t}$, after the translation of *x* using $G_{s\to t}$, it should ideally output the same image.

(2) $$L_{identity}^{s\to t}=E_{x}{}_{∼}{}_{X}{}_{t}[\;|x-G_{s\to t}\left(\;x\right)\;{|}_{1}]\;$$

$$L_{identity}=L_{identity}^{s\to t}+L_{identity}^{t\to s}$$

**LsGAN Loss:** The generator tries to confuse the discriminator into making errors. For example, the generator attempts to generate images that resemble the style of the dataset, whereas the discriminator tries to recognize the generated images. Instead of using the vanilla GAN objective, we used the least-squares GAN [51] objective for regular training.

(3) $$L_{lsgan}^{s\to t}=E_{x}{}_{∼}{}_{X}{}_{t}\left[\;\left(\;D{}_{t}\left(\;x\right)\;\right){\;}^{2}\right]\;+E_{x}{}_{∼}{}_{X}{}_{s}\left[\;{\left(\;1-D{}_{t}\left(\;G_{s\to t}\left(\;x\right)\;\right)\right)}^{2}\;\right]\;$$

$$L_{lsgan}=L_{lsgan}^{s\to t}+L_{lsgan}^{t\to s}$$

**CAM Loss:** Using information from the auxiliary classifiers $\eta {}_{s}$ and $\eta {}_{t}$, provided an image $x\in \{X{}_{s},X{}_{t}\},$$G_{s\to t}$ and $D{}_{t}$ gain insights into where they need to enhance or what makes the most difference between the two domains in the present state:

(4) $$L_{cam}^{s\to t}=E_{x}{}_{∼}{}_{X}{}_{s}\left[\;\log\left(\;\eta {}_{s}\left(\;x\right)\;\right)\;\right]\;+E_{x}{}_{∼}{}_{X}{}_{t}\left[\;\log\left(\;1-\eta {}_{s}\left(\;x\right)\;\right)\;\right]\;$$

(5) $$L_{cam}^{D{}_{t}}=E_{x}{}_{∼}{}_{X}{}_{t}\left[\;\left(\;\eta {}_{D}{}_{t}\left(\;x\right)\;\right){\;}^{2}\right]\;+E_{x}{}_{∼}{}_{X}{}_{s}\left[\;\left(\;\eta {}_{D}{}_{t}\left(\;G_{s\to t}{\left(\;x\right)}^{2}\;\right)\right)\right]\;$$

(6) $$L_{cam}=L_{cam}^{s\to t}+L_{cam}^{D{}_{t}}+L_{cam}^{t\to s}+L_{cam}^{D{}_{s}}$$

**Total Loss:** The final objective is optimized by jointly training the encoders, decoders, discriminators, and auxiliary classifiers. Thus, the final objective function is as follows:

(7) $$L=\lambda {}_{1}L_{Cycle}+\lambda {}_{2}L_{identity}+\lambda {}_{3}L_{lsgan}+\lambda {}_{4}L_{cam}.$$

where $\lambda {}_{1}=1,\lambda {}_{2}=10,\lambda {}_{3}=10,\lambda {}_{4}=1000$.

## 4. Experiments

The details of the experiments are presented in this section. First, we describe the datasets and baselines used in our study. We then describe the results of a qualitative and quantitative comparison. Finally, we develop our model’s ablation study.

### 4.1. Baseline

We compared the proposed method with various models, including U-GAT-IT [1], CycleGAN [11], UNIT [22], CartoonGAN [52], Hneg-SRC [53], and the pre-trained weight of Stable Diffusion v1.5 [54] obtained from runwayml. It is worth noting that our analysis was not limited to image-to-image translation models exclusively. In order to accurately assess the effectiveness of our method, we also incorporated advanced state-of-the-art diffusion models as comparative baselines. While the baseline methods lacked the capability to generate images across various resolutions, we addressed this limitation by conducting separate training for each specific resolution. However, it is important to note that this resolution-specific training was not applied to the pre-trained weight of Stable Diffusion v1.5 obtained from runwayml.

For the image translation outcomes using Stable Diffusion v1.5, we employed specific settings, including a seed value of 620,974,597, CFG (classifier-free guidance) Scale set to 7, Euler sampling method, and a denoising strength of 0.75. By using the prompt “a face of a girl”, we successfully converted an anime-style image into a realistic human picture. Similarly, when provided with the prompt “a face in anime style”, we were able to transform a human picture into an anime character.

### 4.2. Dataset

**Figure 2:**
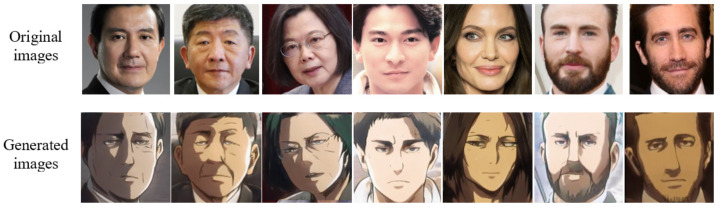
Translation of human images into the animation characters in Attack on Titan.

**AnimeFace2CoserFace.** We collected around 30,000 images of coser people dressed like anime characters from Flickr and around 50,000 images of anime girls from Pixiv, a Japanese open-source website. We used a pre-trained MXnet model from the light anime face detector repository to crop the face in the image and resized it to 1024 × 1024.
**Anime2Coser.** We gathered around 20,000 images of coser people from Flickr and 25,000 bust photos of anime characters from Pixiv. We used a pre-trained MXnet model from the light anime face detector repository, expanding a 30% larger detection area to crop bust photos and resize them to 1024 × 1024.
**AttackOnHuman.** We collected around 30,000 face images from Attack on Titan, a Japanese dark fantasy anime television series, and cropped the faces in the videos using the light anime face detector. In addition, we used around 15,000 cropped images from the Flickr Faces-HQ (FFHQ) dataset for the human domain dataset. Finally, we resized all the images to 512 × 512. The generated results are shown in Figure 2.

### 4.3. Training

Due to the progressive training, we input different image sizes for each training step and gradually increased the size of the images from 64, 128…, to 1024. For each training step, we resized the images to (30 + training size) × (30 + training size) and cropped them into training size × training size as input. All the parameters of the proposed method were initialized from a zero-centered normal distribution with a standard deviation of 0.02, and we set the hyperparameters $\lambda {}_{1}=1,\lambda {}_{2}=10,\lambda {}_{3}=10,$ and $\lambda {}_{4}=1000$ for the final objective function. The Adam optimizer was utilized with β1=0.5 and β2=0.999. We trained the model for 200 epochs, fixed the learning rate at 0.0005 until epoch 50, and decayed it along with the ratio 1−epoch/(total_epoch) until the end of the training process. During each training step, the batch size changed, starting with 8 for 64 × 64 resolution, 4 for 128 × 128, and finally fixed at 2 for resolutions larger than 128 × 128. We implemented our method using the PyTorch Library and used 8 Tesla V100 GPUs to train the modal.

### 4.4. Qualitative Evaluation

**Figure 3:**
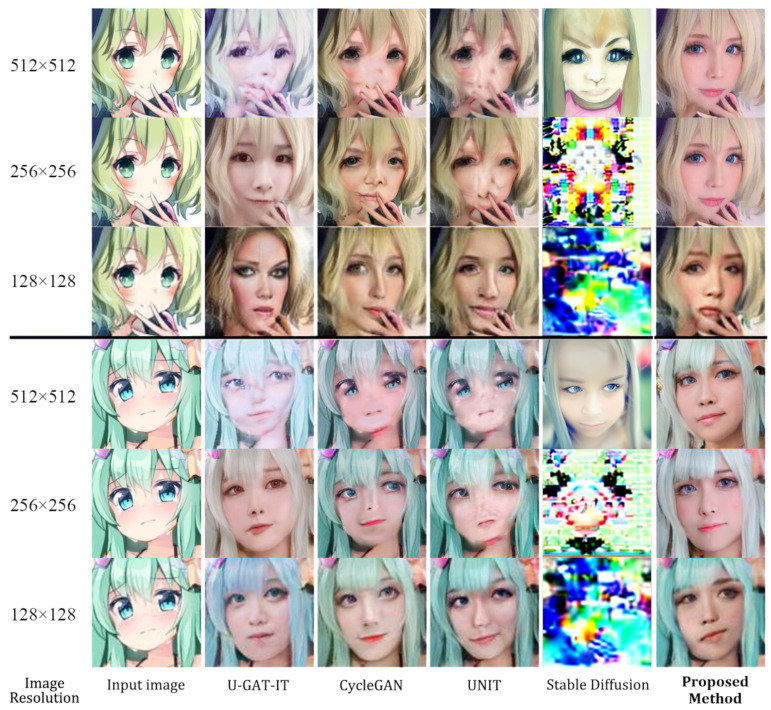
Anime character translation into a coser.

**Figure 4:**
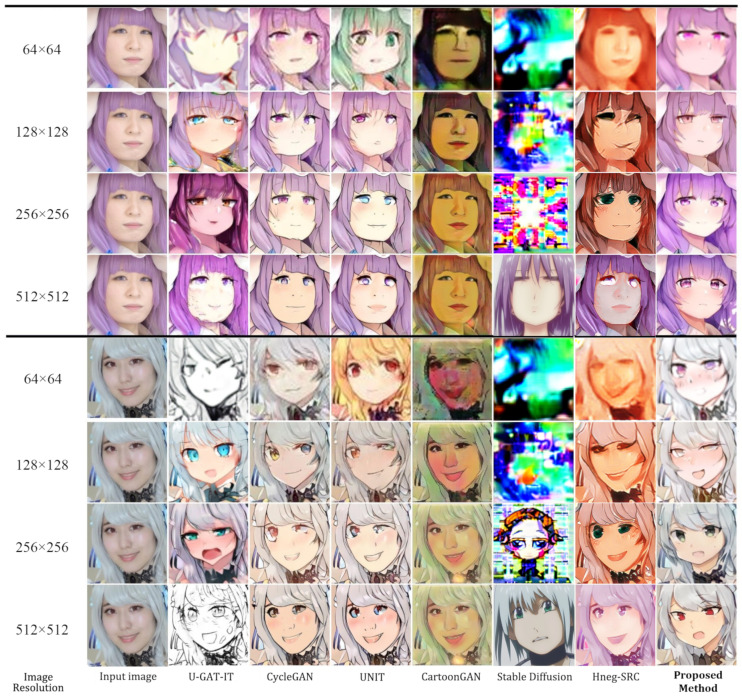
Coser translation into an anime character. Compared to U-GAT-IT, the results generated by our model preserved the color, local structure, and global structure, with fewer glitches.

Figure 3 compares the qualitative results of the proposed method with those of UNIT, CycleGAN, U-GAT-IT, and Stable Diffusion. From the comparison, it is clear that our approach demonstrates a higher quality of image translation than the other methods. In contrast, the other methods failed to transfer facial features and generated many blurs. Furthermore, our approach could translate the details into another domain, which seems more realistic in the target domain. The model can learn features from different scales with the effective PRO-DS blocks and PRO-US blocks, allowing the generator to capture the image details and fully transfer them, therefore outperforming the previous method. As shown in Figure 4, with the large size of the generated image, our method generated a better image when processing 512 × 512 images due to successfully translating the anime face into a human face, with local and global structures preserved. However, despite the small image size, our method produced images with distortions and incongruent facial changes, similar to the other approaches. It is worth mentioning that we used the same model to generate images of different sizes, whereas the other methods followed different sizes of image models to train their respective models independently.

**Figure 5:**
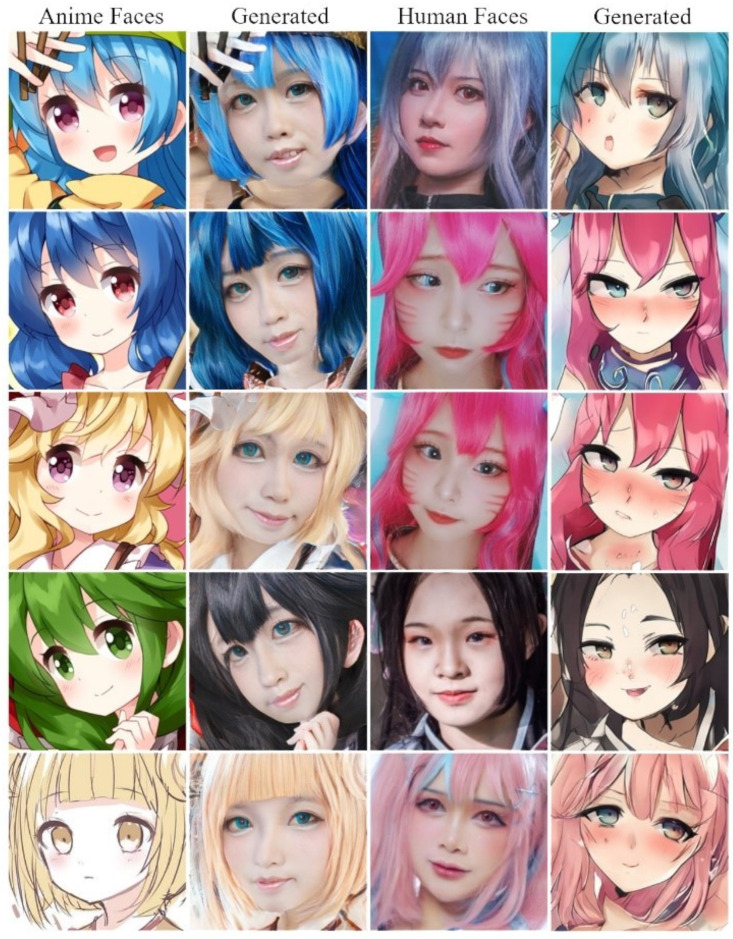
The proposed PRO-U-GAT-IT method can generate 1024 × 1024 images from 256 × 256 images, and it can translate the face crop of the image and a large area of the human face.

On the contrary, to translate the human face into an anime face, as shown in Figure 4, we added CartoonGAN for comparison. Even though CartoonGAN has excellent translation quality, the generated images were different from the anime ones, as it was like adding filters to the original images. Furthermore, CycleGAN generated images with poor details, such as the eyes, and both UNIT and U-GAT-IT failed to generate corresponding colors correctly from the original images. Based on progressive feature learning, our method generated a larger image than the input image, as shown in Figure 5, generating 1024 × 1024 size images from 256 × 256 images.

In addition, Hneg-SRC demonstrated an ability to generate anime-like textures with remarkable quality. However, it fell short in terms of translating the overall features of the image. Rather than transforming the shape and appearance of the human image, Hneg-SRC primarily focused on altering the texture while preserving the original shape. In contrast, the pre-trained weights of Stable Diffusion v1.5 excelled in producing impressive image quality at a resolution of 512 × 512, as the model was explicitly trained at that specific resolution. It exhibited commendable performance in anime-to-human translation tasks, although occasional artificial glitches still arose. However, it is crucial to note that the model’s effectiveness diminished when confronted with resolutions lower than 512 × 512, as it was constrained by its training limitations and was unable to generate meaningful images in such cases.

To test the translation capability of the model, we collected pictures of public figures online and translated them into animation with a distinct style. Our method generated higher-quality images and retained the expressions from the original photos, matching the animation’s unique light and dark style, resulting in a more realistic animation style.

**Figure 6:**
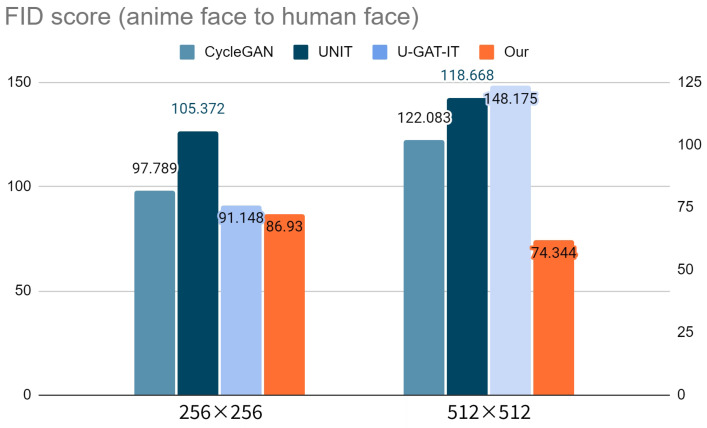
Visualization of the FID scores for anime-to-human faces with two sizes of generated images.

**Figure 7:**
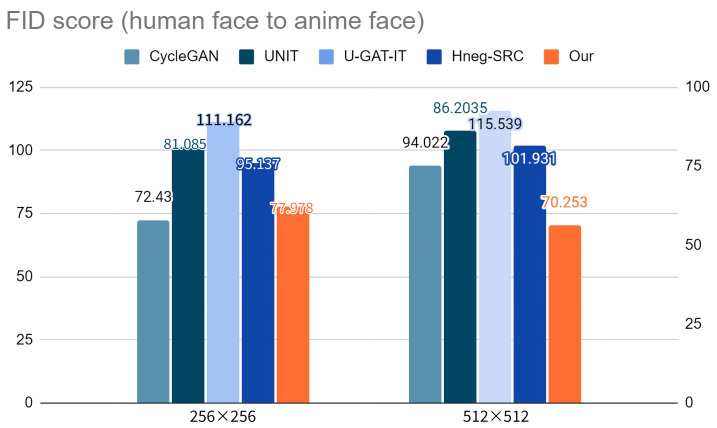
Visualization of the FID scores for human-to anime-faces with two sizes of generated images.

We utilized the Fréchet inception distance score [55], commonly used in image generation tasks, for the quantitative evaluation. Table 1 and Table 2 summarize the FID score for each model. Figure 6 and Figure 7 show a visual representation of the FID scores for the four models, where a lower FID represents better image quality. The results show that our method generated the lowest FID score, even for large images.

To determine whether the images generated by our proposed method are realistic, we conducted human voting using Google Forms. The voting form consisted of two sections. The first section contained around 27 questions. In each question, there were four images, including one generated image. A total of 16 questions showed the image generated by our proposed method, and 11 questions showed the image generated by U-GAT-IT. First, we asked our subjects to distinguish between the produced image and the other three images. Next, we asked our participants to select the best-generated image among those generated by CycleGAN, UNIT, U-GAT-IT, and our proposed method.

As shown in Table 3, the images generated by our proposed model received lower correctness from human voting, thus making them appear more realistic to human readers than those generated by U-GAT-IT. The second section shows that 61% of the images generated by our method were voted as the best among the four images generated by CycleGAN, UNIT, and U-GAT-IT, as shown in Table 4.

**Figure 8:**
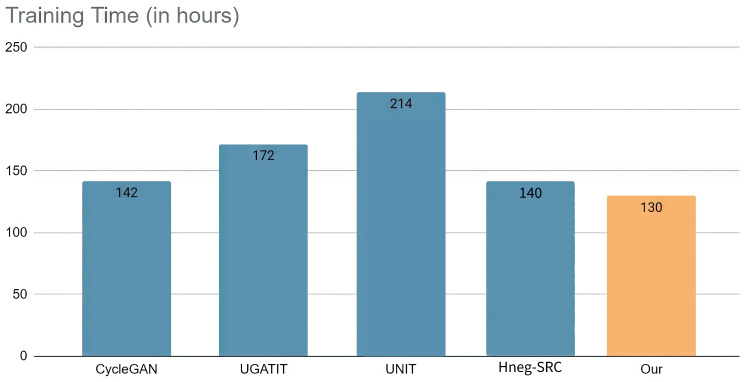
In the comparison of the training times, we can see that due to progressive training, our model trained faster than the other models during the 512 × 512 translation model training.

**Figure 9:**
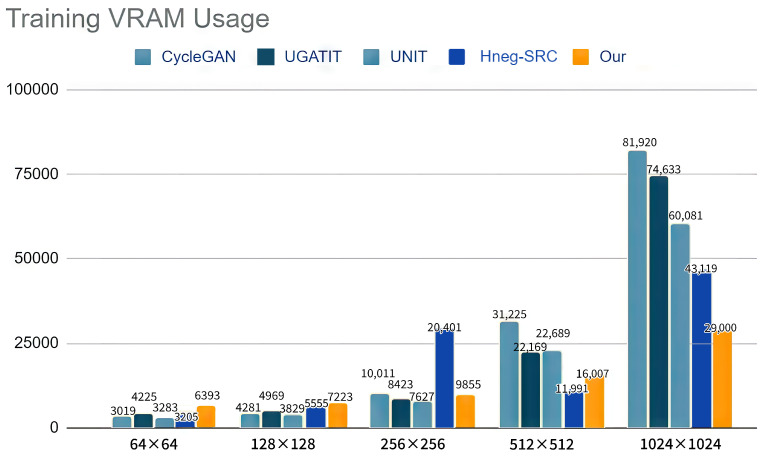
Visualization of the VRAM usage of each method during training with different image sizes.

Aside from reducing training speed and Video Random-Access Memory (VRAM) usage, our method also improved image quality. We compared our method with CycleGAN, U-GAT-IT, UNIT, and Hneg-SRC with a 512 × 512 resolution, and the results are shown in Figure 8 and Figure 9, and Table 5. Using 1,500,000 iterations, we trained CycleGAN, U-GAT-IT, UNIT, and Hneg-SRC. In the proposed method, we trained using a 64 × 64 resolution for 200,000 iterations, a 128 × 128 resolution for 300,000 iterations, a 256 × 256 resolution for 400,000 iterations, and a 512 × 512 resolution for 600,000 iterations. Our method achieved the shortest training time in terms of speed, which was about 25% faster than U-GAT-IT. However, our method did not trade space for time. Therefore, instead of reducing VRAM usage, we reduced the training time. Despite using more VRAM when training on small images, our VRAM usage dropped to 70–50% after reaching the 512 × 512 image size, and our VRAM usage was only 30% compared to CycleGAN at the 1024 × 1024 image size, which was lower than the other methods.

During our experiments, we observed a notable reduction in VRAM usage when training Hneg-SRC at a resolution of 512 × 512, with the total VRAM consumption averaging around 11 GB. However, when training at a higher resolution of 1024 × 1024, the VRAM usage significantly increased to approximately 40 GB. Based on our observations, we speculate that the official implementation of Hneg-SRC prioritizes training speed optimization at a 256 × 256 resolution, where the VRAM usage is around 20 GB.

## 5. Ablation Study

**Figure 10:**
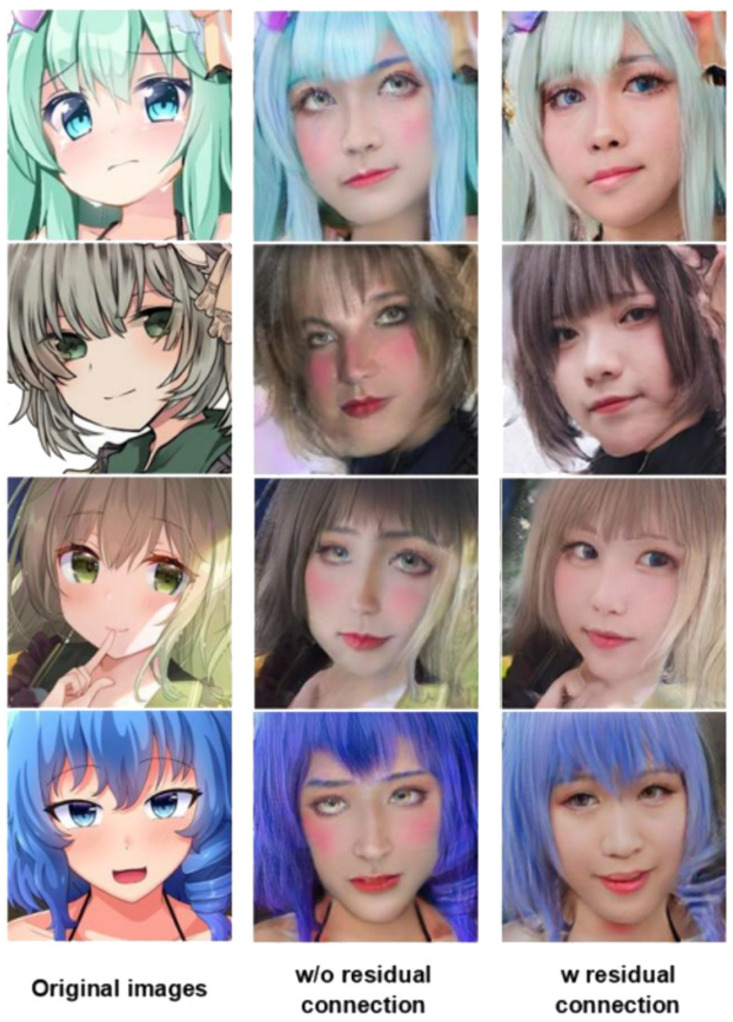
The result of using a residual connection is better quality. In addition, the image generated with a residual connection looks better due to fewer artificial glitches.

Figure 10 shows the results of the ablation study. We conducted an ablation study for the residual connection in the PRO-US block of the generator’s decoder. Adding a residual connection in the PRO-US block made the generated images more colorful and stable, and they were better than those without the addition of a residual connection in the progressive decoder blocks.

## 6. Conclusions

In this paper, we proposed an unsupervised image-to-image translation method called PRO-U-GAT-IT by dynamically adding progressive blocks, which can produce higher-resolution images with better image quality and detail translation. This research has potential applications in various fields, such as online virtual streaming, the anime industry, and data augmentation scenarios that require higher resolutions. In our experiments, our method outperformed existing state-of-the-art models for unsupervised image-to-image translation tasks. Our framework adopts a progressive learning scheme, where different scale features are learned separately in each layer. By employing these advancements, we significantly reduced VRAM usage, enhanced training speed on the same device compared to the other methods we evaluated, and achieved successful image translation at higher resolutions. However, we observed a noticeable decline in the model’s performance at lower resolutions due to the small patch size of our discriminator. Additionally, the model often generated artificial artifacts in its output. Our future work aims to address these issues and improve the overall performance of the model. The realistic outputs of PRO-U-GAT-IT raise concerns regarding potential misuse and its impact on different industries. It is important to take measures to address the creation of deceptive content, protect intellectual property, and ensure responsible use to mitigate any negative consequences.

## Figures and Tables

**Table 1:** FID scores for anime-to-human faces with two sizes of generated images.

Image Size	CycleGAN	UNIT	U-GAT-IT	Our Model
256 × 256	97.789	105.372	91.148	**86.930**
512 × 512	122.083	118.668	148.175	**74.344**

**Table 2:** FID scores for human-to-anime faces with two sizes of generated images.

Image Size	CycleGAN	UNIT	U-GAT-IT	Hneg-SRC	Our Model
256 × 256	**72.430**	81.085	111.162	95.137	77.978
512 × 512	94.022	86.205	115.539	101.931	**70.253**

**Table 3:** Percentages of generated images distinguished by human readers. A lower percentage of correctness implies that the images are more realistic, which means that the images are harder to distinguish.

	U-GAT-IT	Our Model
Correctness of Human Face to Anime Face	63.04%	**37.25%**
Correctness of Anime Face to Human Face	58.40%	**48.51%**

**Table 4:** Percentages of the best-generated images selected by human readers out of four generated images.

	CycleGAN	UNIT	U-GAT-IT	Our Model
Generated image accuracy	5.4%	10.7%	22.9%	**61%**

**Table 5:** VRAM usage of each method during training with different sizes of images.

Image Size	CycleGAN	UNIT	U-GAT-IT	Hneg-SRC	Our Model
64 × 64	3203 MB	**3019 MB**	4225 MB	3205 MB	6393 MB
128 × 128	4281 MB	**3829 MB**	4969 MB	5555 MB	7223 MB
256 × 256	10,011 MB	**7627 MB**	8423 MB	20,401 MB	9855 MB
512 × 512	31,225 MB	22,689 MB	22,169 MB	**11,991 MB**	16,007 MB
1024 × 1024	81,920 MB	60,081 MB	74,633 MB	43,119 MB	**29,000 MB**

## Data Availability

Publicly available datasets were analyzed in this study. This data can be found here: https://github.com/NVlabs/ffhq-dataset and https://gwern.net/danbooru2021. Implementation details are available at: https://github.com/sam3u7858/pro-ugatit.
